# A Convolutional Autoencoder Topology for Classification in High-Dimensional Noisy Image Datasets

**DOI:** 10.3390/s21227731

**Published:** 2021-11-20

**Authors:** Emmanuel Pintelas, Ioannis E. Livieris, Panagiotis E. Pintelas

**Affiliations:** 1 Department of Mathematics, University of Patras, 26500 Patras, Greece; pintelas@math.upatras.gr; 2 Core Innovation and Technology O.E., 11745 Athens, Greece; livieris@upatras.gr

**Keywords:** convolutional autoencoders, dimensionality reduction, deep learning, convolutional neural networks, computer vision, image classification

## Abstract

Deep convolutional neural networks have shown remarkable performance in the image classification domain. However, Deep Learning models are vulnerable to noise and redundant information encapsulated into the high-dimensional raw input images, leading to unstable and unreliable predictions. Autoencoders constitute an unsupervised dimensionality reduction technique, proven to filter out noise and redundant information and create robust and stable feature representations. In this work, in order to resolve the problem of DL models’ vulnerability, we propose a convolutional autoencoder topological model for compressing and filtering out noise and redundant information from initial high dimensionality input images and then feeding this compressed output into convolutional neural networks. Our results reveal the efficiency of the proposed approach, leading to a significant performance improvement compared to Deep Learning models trained with the initial raw images.

## 1. Introduction

Nowadays, convolutional neural networks (CNNs) have considerably flourished mainly because they have shown noticeable classification performance in image classification and computer vision tasks [1,2]. However, robustness and stability are some major problems in which Deep Learning (DL) models are prone, since it is proved that they can be fooled even by a tiny amount of perturbation, exhibiting poor and unreliable performance in these cases [3,4].

Moreover, in Machine Learning (ML) image classification tasks when dealing with high-dimensional data, which usually contain a lot of redundant information and noise, the reliable knowledge feature extraction procedure deteriorates [5]. The extraction of only the most important features compresses the initial feature space, leading to a stable and robust latent image representation [2,5,6]. Thus, it is necessary to capture only the most relevant information.

Training a supervised DL model with high dimensionality and low-quality image data can lead to overfitting and/or unstable behavior, especially when the training instances are limited or unbalanced. In other words, small pixel changes can lead the model to change its predictions, which implies that it has not exploited the information in the training data, and it exhibits poor and inefficient performance [7]. Additionally, it is worth highlighting that another significant problem is that the higher the dimension of the input images, the more the network is affected by the presence of noise, even if the amount of noise is small. By taking into consideration these difficulties and constraints, the application of a preproccessing step, which will attempt to reduce the noise in the image data while simultaneously reduce their dimension is considered essential for improving the performance of the DL model.

A traditional approach for image denoising is the transformation of the image from pixel intensities into another representation in order to capture the image’s statistical regularities more easily and effectively [8]. In the literature, a variety of approaches have been proposed for image denoising such as Gaussian scale mixture (GSM) models [9,10] and the more elegant Markov random fields (MRF)-based methodologies [11,12]. Nevertheless, the main disadvantage of these approaches is the considerable computational cost for parameter estimation and the fact that their values significantly affect the denoising performance quality. For example, naive methods of learning MRF-based models require the calculation of the partition function as well as a normalization factor which is generally intractable for high image dimensions [8]. To this end, a significant amount of research has been devoted to approximate MRF learning and inference techniques, which are usually computationally inefficient; thus, the parameters estimation is a significantly hard task. In addition, even if a traditional method is successfully applied, the noise in the images will be considerably reduced in the best-case scenario, but not totally removed. This implies that the high dimension of the input images, together with any amount of noise, will probably lead to unsatisfying performance, although the amount of noise may be small.

Convolutional autoencoder (CAE) [7] models constitute neural-network-based models, which have been proposed for dimensionality reduction and representation learning in a variety of tasks [13,14,15]. These models avoid the computational cost drawback of image denoising by posing the task within the statistical framework of regression, which constitutes a more tractable computation; thus, it permits greater representational power than density estimation [8]. More specifically, the process of image denoising with a CAE model can be formulated as a learning problem of training the model; therefore, the parameter estimation is performed by a modification of the well-known backpropagation algorithm [16]. The novelty in CAE for image processing is the utilization of convolutional layers which are able to create more abstract representations of the initial inputs by removing noise and redundant information. Therefore, these layers have been characterized as one of the major frontiers in deep learning and image analysis [7].

The utilization of convolutional layers allows CAEs to filter out noise and create robust and stable feature representations [5,17] while simultaneously reducing the input dimension size, making them suitable for dealing with high-dimensional noisy images. It is worth mentioning that an attractive property of CAEs over traditional dense autoencoders for image processing is that generally a huge loss of information is noticed when stacking and slicing the data. Instead of stacking the data as in classical autoencoders, the convolutional layers of CAEs are able to efficiently retain the spatial information of the input image data and gently extract information. In other words, CAEs can learn compressed image latent representations [5,17,18], therefore preserving the spatial locality of the input in a manner similar to other CNNs [19].

In this work, we propose an ML topology for performing efficient and robust classification in high-dimensional and noisy input data images. As a first preprocessing step, we utilize a CAE model in order to compress and filter out noise and redundant information, maintaining also the spatial feature characteristics from the initial image data and capturing only the most relevant and useful feature information. As a result, the initial high-dimensional input is transformed via the CAE into a compressed and compact 2D spatial image representation. In the second step, the CAE outputs are fed into a powerful pre-trained image classification model in order to successfully address classification tasks.

The main contribution of this research lies in proposing and implementing CAE as a general unsupervised learning data preprocessing method for creating robust and compressed feature representations. The rationale behind our approach is to improve state-of-the-art DL models to perform stable and accurate predictions on classification tasks when high-dimensional and noisy image input datasets are involved. The aim of this work is to tackle the problem of Deep Learning models being vulnerable in noise and redundant information tied into the raw input images, especially from high-dimensional inputs, leading to unstable and unreliable predictions. To this end, our approach is based on exploiting the advantages of CAEs as an efficient pre-processing noise filtering and compression method in order to improve CNN models in terms of robustness and accuracy when dealing with noisy high-dimensional inputs.

In order to validate the efficacy and the efficiency of the proposed method, we performed extensive experimental simulations utilizing high-dimensional image datasets concerning three different application domains. The first dataset concerns the problem of plant disease detection, the second concerns skin cancer detection, and the third DeepFake detection.

The rest of this paper is organized as follows: in Section 2, we present the state of the art image classification models and our proposed topology, while in Section 3, we present the technical details concerning the utilized datasets. Section 4 demonstrates our experimental results, and finally, Section 5 sketches our conclusive remarks and the future directions.

## 2. Materials and Methods

### 2.1. Related Work

During the last decades, a remarkable number of methodologies have been proposed in order to remove noise and redundant information from input images for creating robust and efficient image representations. Tian et al. [20] presented an excellent review regarding conventional machine learning methods and deep learning technologies for image denoising. The authors presented the most decent works proposed in the literature, focusing on the advantages of each approach. Finally, they discussed some promising research directions for image denoising based on deep learning technologies. In general, image denoising approaches can be separated into the traditional ones, such as GSM models and MRF-based methodologies, and the most elegant—the CAE-based approaches.

Portilla et al. [10] proposed a new methodology for noise removal from digital images which was based on a local GSM model in an overcomplete oriented pyramid representation. In their proposed methodology, they computed the full optimal local Bayesian least squares solution (LSS), as opposed to first approximating the local variance, and then utilized it to estimate the coefficient. Additionally, we utilized the vectorial form of the LLS solution in order to exploit all the information provided by the covariance modeling of signal and noise. The authors provided empirical evidence that these enhancements considerably improve the denoising performance.

Tappen et al. [11] presented a new approach for training a Gaussian Conditional Random Field (GCRF) model for image denoising, which is able to outperform the non-convex Field of Experts model. The rationale behind their approach of focusing on discrete-valued and non-convex MRF models was that GSM models tend to over-smooth images and blur edges. An advantage of the GCRF model is that its parameters can be optimized efficiently on relatively large images. Based on their experimental analysis, the authors stated that their proposed approach constitutes an attractive option for image and vision processing applications.

Barbu [21] proposed an interesting approach in which it was demonstrated that the process of training an MRF/CRF model together with a very fast inference algorithm could offer promising results relative to both speed and accuracy. The key idea of the proposed approach was that a validation set can be utilized to estimate the generalization performance of the trained system. Their experiment was performance on 256 × 256 images which presented that the proposed approach obtained an improved performance as well as a 1000–3000 times speedup compared to the state-of-the-art Field of Experts MRF trained with contrastive divergence.

Zhang et al. [22] proposed a novel Gaussian mixture Markov random field model (GM-MRF) which can be efficiently utilized as a very expressive prior model for image denoising and reconstruction. The proposed method forms a global image model by merging together individual GSM mixture models for image patches. Furthermore, the authors analytically presented a framework for computing MAP estimates with the GM-MRF model through the construction of exact surrogate functions. Their experimental analysis included a demonstration of the efficiency of their approach for denoising of dual-energy CT images.

Chen et al. [23] proposed a new framework which was based on a convolutional autoencoder model for creating unsupervised representations for images of lung nodule. More specifically, their approach was composed by a two-stage training procedure: at the first phase, the CAE was trained in an unsupervised way utilizing unlabeled data for image features learning; in the second phase the CAE is merged with a dense neural network and the resulting model was trained in a supervised way utilizing labeled data. An attractive property of the proposed approach comparing to a supervised one, is that it requires a small amount of labeled data for efficient feature learning applied in classification tasks. Additionally, the authors provided evidence that their proposed methodology can be extended for similarity measurement tasks of lung nodules images.

Seyfioğlu et al. [24] proposed a three-layer CAE topology for radar-based classification of similar aided and unaided human activities. After the unsupervised training procedure of the CAE, the decoder was removed, and it was substituted by dense layers and an output softmax layer. The develoved convolutional-based classification model was then trained in a supervised way. Their experimental analysis showed that their proposed methodology was superior compared to other deep learning classification models, support vector machines, extreme gradient boosting and random forest.

The main difference of our proposed approach compared to the previous state-of-the-art approaches lies in the fact that a CAE is trained for filtering out noise and creating robust and stable feature representations while simultaneously reducing the input image dimension size. In the sequence, the output of the encoder is utilized for developing a training set to fit a powerful pre-trained image classification model. The rationale behind our approach is to enhance the predictive power of pre-trained neural network classification model by developing a higher quality training dataset. For this purpose, we utilized a CAE for simultaneously compressing an image and filtering out noise and redundant information while also maintaining the spatial feature characteristics from the initial image data.

### 2.2. State-of-Art Pre-Trained CNN Classification Models

Large Deep Learning models trained on over millions of images, composed by a large variety of various CNN architectures, topologies such as VGG and ResNet, are considered as the mainstream approaches for addressing image classification applications [1,2]. In fact, these networks are utilized as pre-trained feature extraction models transferring their knowledge into new small non-trained networks (main transfer learning approach) in order to specialize in new specific image classification problems.

VGG [19] adopted its name by the team Visual Geometry Group at the University of Oxford and is applied in computer vision tasks. This deep neural network is constituted by multiple (3×3) convolution filters, which are proved to be more efficient compared to its prior network AlexNet. On the other hand, AlexNet was composed by kernel-sized filters (11 and 5 in the first and second layer, respectively).

ResNet [25], also called Residual Network, is a Deep Learning model which utilizes identity connections in order to address the degradation problem which is caused by very large network depths (such as over 18 layers). In particular, these connections take the input directly to the end of each residual block, while each residual block is constituted by 3 × 3 and 1 × 1 convolution filters.

DenseNet [26] constitutes an updated version of ResNet and is implemented using dense blocks, which connect each layer to every other layer in a feedforward way. The main advantages of utilizing these blocks are feature reuse, implicit deep supervision, and parameter efficiency.

MobileNet [27] is a computationally efficient state of the art CNN topology designed for application mainly to mobiles. MobileNet’s topology is based on an inverted residual structure, while the input and output of the residual block are thin bottleneck layers in contrast to classic residual networks.

### 2.3. Convolutional Autoencoders

Convolutional autoencoders (CAEs) are unsupervised dimensionality reduction models composed by convolutional layers capable of creating compressed image representations [28]. In general, CAEs are mainly utilized for reducing and compressing the input dimension size, removing noise while simultaneously keeping all useful information and extracting robust features [5,17,18].

The main difference between convolutional AE and traditional AE is the utilization of convolutional layers. It is worth mentioning that these layers are characterized by their attractive property of extracting knowledge and learning the internal representation of image data.

**Figure 1:**
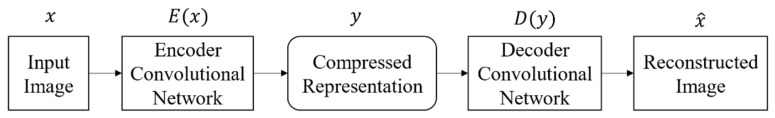
General pipeline of a CAE model.

More specifically, CAEs are composed by two CNN models, the *Encoder* and *Decoder*, as presented in Figure 1. The Encoder is mainly used for encoding the initial input image into a latent representation which has lower dimension. On the other hand, the Decoder is responsible for reconstructing the compressed latent representation creating an output image being as much similar with the initial one.

Mathematically, let $x=X_{initial}^{M\times H\times W\times 3}$ denote the initial input image dataset, where *H*, *W* are the number of pixels corresponding to the Height and Width of every image *X*, and *M* is the number of samples. Additionally, *E* and *D* denote the Encoder and Decoder, respectively. Then, the encoded representation $y=X_{compressed}^{M\times h\times w\times 3}$, where *h* and *w* are the width and depth dimensions of the compressed spatial 2D representation, and the decoded reconstruction $\hat{x}=X_{initial}^{M\times H\times W\times 3}$ consist of the output of the encoder and decoder, respectively, that is:

$$\left\{\begin{array}{ccc} y & = & E(x)\\ \hat{x} & = & D(y) \end{array}\right.$$

The performance of the convolutional autoencoder can be measured by the reconstruction error $e^{CAE}$, which is defined by:

$$e^{CAE}=L_{CAE}\left(\left({\hat{x}}^{\left(k\right)}\right),x^{\left(k\right)}\right)$$

The function $L_{CAE}$ denotes a measurement of difference such as the widely used square Euclidean distance defined as:

$$L_{CAE}\left({\hat{x}}^{\left(k\right)},x^{(k})\right)=\frac{1}{2}{∥{\hat{x}}^{\left(k\right)}-x^{\left(k\right)}∥}^{2}.$$

Then, the cost function in its general form can be formulated as follows:

(1) $$J_{CAE}=\frac{1}{M}\sum_{k=1}^{M}L_{CAE}\left(D\left(E\left(x^{\left(k\right)}\right)\right),x^{\left(k\right)}\right)$$

By minimizing the cost function $J_{CAE}$, we attempt to find the optimal weight parameters for the convolutional autoencoder.

In our implementation, the cost function (1) was minimized utilizing Adam optimization algorithm [29], and the value of the learning rate was set to $10^{-3}$.

### 2.4. Proposed Topology

**Figure 2:**
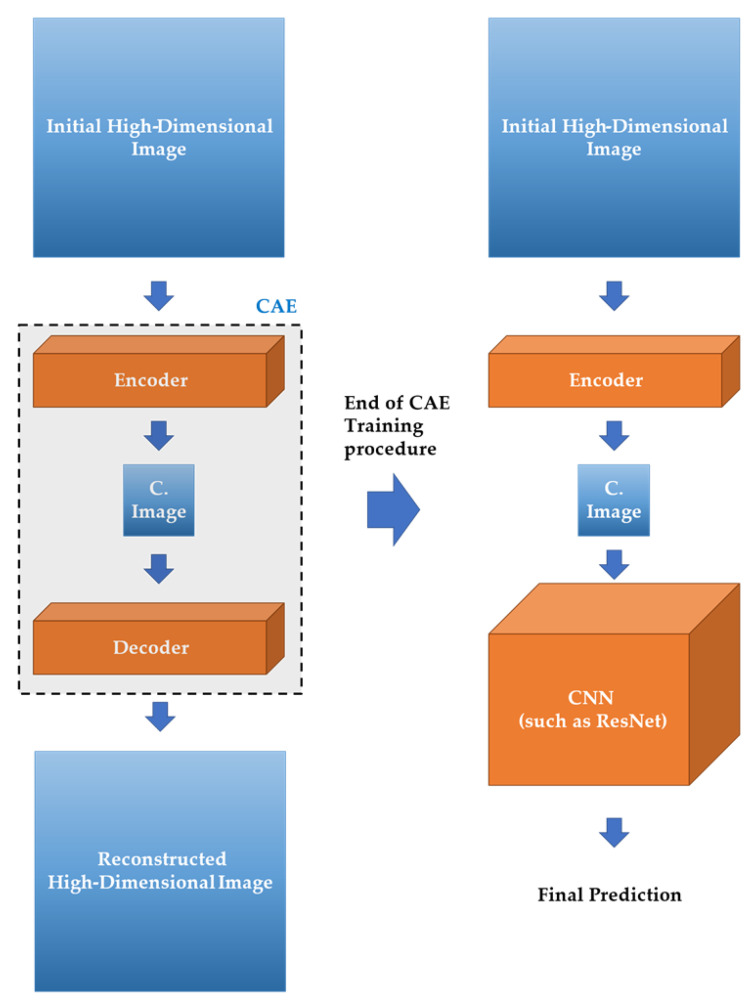
Main pipeline of the proposed CAE-CNN topology.

Figure 2 presents the main pipeline of the proposed convolutional autoencoder–convolutional neural network (CAE-CNN) topology. In our approach, initially a CAE is trained with the initial training dataset. When the CAE finishes its training procedure, then the decoder component is discarded, while the encoder is used for compressing the initial high-dimensional image dataset into a compressed image dataset. Finally, the output of the CAE’s encoder (compressed image dataset) is used for feeding and training a CNN classification model, such as ResNet, VGG, etc.

In the sequel, let us denote *C* as the CNN classification model and $l=\{l_{1},l_{2},\cdots ,l_{N}\}$, where $l_{i}\in \left\{0,1\right\}$∀i∈N, as the target output of N total classes with respect to the classification problem. The initial training dataset *x* is transformed via the encoder *E* into an encoded compressed 2D representation *y*. The raw output $\hat{l}=\left\{{\hat{l}}_{1},{\hat{l}}_{2},\cdots ,{\hat{l}}_{N}\right\}$ of the CNN classification model is given as follows:

$$\hat{l}=C\left(y\right)$$

The performance of the CNN classification model can be measured by the reconstruction error:

$$e_{CNN}=L_{CNN}\left({\hat{l}}^{\left(k\right)},l^{\left(k\right)}\right)$$

The function $L_{CNN}$ denotes a measurement of difference such as the widely used Cross Entropy loss function [30]. Then, the cost function in its general form can be formulated as follows:

$$J_{CNN}=\frac{1}{M}\sum_{k=1}^{M}L_{CAE}\left(D\left(E\left(l^{\left(k\right)}\right)\right),l^{(}\left(k\right))\right)$$

Finally, by minimizing the cost function $J_{CNN}$, we obtain the optimal weight parameters for the CNN classification model with respect to the classification task.

### 2.5. Proposed Convolutional Autoencoder’s Architectural Design

**Figure 3:**
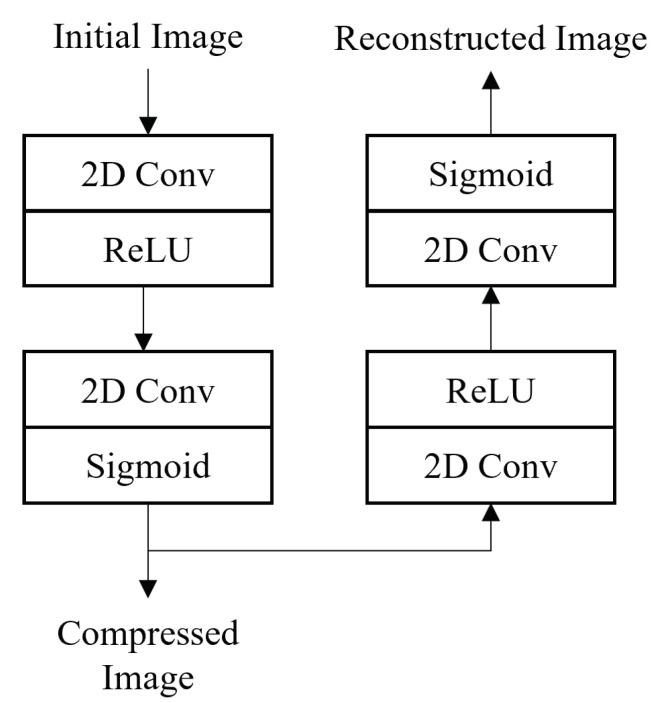
Architectural presentation of our CAE topology.

**Figure 4:**
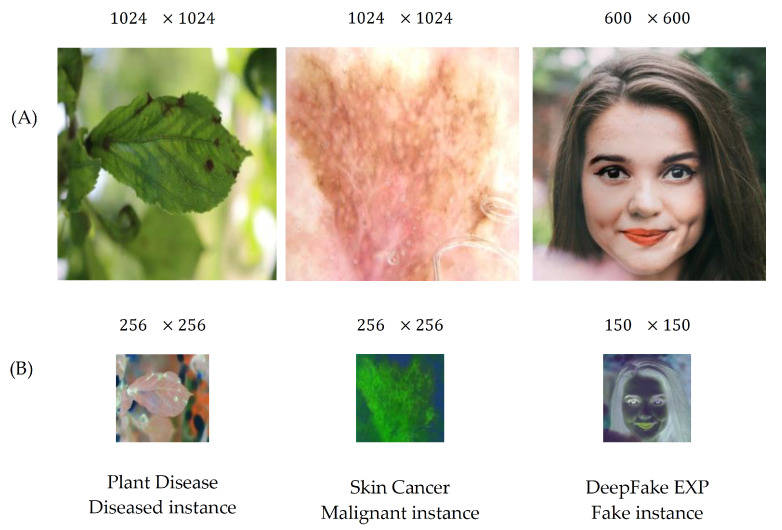
(**A**) Initial high-dimensional image. (**B**) Compressed image using CAE.

Figure 3 presents the proposed CAE topology of our architecture, while Figure 4 presents the compressed image output of the CAE of some examples regarding the three utilized image datasets. Finally, Table 1 presents in a detailed way the CAE parameters’ settings configurations setup. The proposed CAE has a symmetric architecture with four batches of 2D convolutional and deconvolutional layers followed by a Rectified Linear Unit (ReLU) activation function. The deconvolution [31] (also called as transposed convolution) is performing the reverse operation of the convolutional layer. In particular, it maps the input from a low-dimensional space to a high-dimensional one.

More specifically, the raw input image with dimensions H×W×3 is fed into the first layer (2D Conv1-ReLU1), which is also the CAE’s Encoder’s input and creates 32 down-sampled spatial feature maps of dimensions H/2×W/2, utilizing a 4×4 kernel size with 32 filters. Subsequently, this output is fed into the second layer (2D Conv2-ReLU2), which is the Encoder’s output and creates the compressed image representation of dimensions H/4×W/4×3, utilizing a 2×2 kernel size with 3 filters. Since the first layer’s output feature maps have a lower dimensional size comparing to the input image, it is reasonable to utilize a smaller kernel size in the second layer. Similarly, the third and fourth layers (2D Deconv3-ReLU3, 2D Deconv4-ReLU4) of CAE’s decoder component perform in a symmetric way the reverse operation of this of the encoder’s.

## 3. Case Study Applications/Datasets

Next, we present the characteristics of the datasets, which were utilized in this study in order to evaluate the efficiency of the proposed architectural topology. The first application concerns the problem of plant disease detection, the second concerns the skin cancer detection, and the third the DeepFake detection.

These three application domains have attained very high interest in the last years [32,33,34,35,36,37,38,39] for many different reasons. More specifically, regarding the plant disease problem, due to the massive agriculture improvement, it is necessary to automate plant disease detection by using technologies such as air drones and cure ill plants fast and accurately [40]. Regarding skin cancer detection, the fast recognition and treatment in its earliest stages is crucial for its treatment and curing process. The DeepFake faces image detection problem recently has attained very high interest [33,34,35,36,37,38,39]. The recent invention and the continuous development of the Generative Adversarial Networks (GANs) [40] technology has made possible the generation and the creation of high quality and extremely realistic fake images and videos being very hard even for experts to recognize them. These fake images/videos can be extremely harmful for human rights, especially when the deepfakes are used maliciously as a source of misinformation, manipulation, and harassment.

**Plant disease**. Concerning the plant disease detection problem, the utilized training dataset is composed of 464, 81, 559, and 532 of “*healthy*”, “*multiple-disease*”, “*rust*”, and “*scab*” labeled plants, respectively, while the testing dataset consists of 52, 10, 63, and 60 of “*healthy*”, “*multiple-disease*”, “*rust*”, and “*scab*” labeled plants, respectively. All images had initial resolution of 1024×1024. This dataset is available at: https://drive.google.com/drive/folders/12DIF3KF-ZOnpBzsA6inRRjNNOBLqJhhG (accessed on 14 November 2021).

**Skin cancer**. This dataset was obtained by ISIC (International Skin Imaging Collaboration, https://www.isic-archive.com (accessed on 14 November 2021)), which aims to assist in reducing melanoma mortality through the application of digital skin cancer imaging. The utilized training dataset consists of 1754 and 178, while the testing dataset consists of 168 and 22 of “*Benign*” and “*Malignant*” diagnosed patients, respectively. All images had initial resolution of 1024×1024.

**DeepFake experts**. Concerning the DeepFake detection problem, we utilized a balanced dataset created by expert photoshop designers, conducted by the Computational Intelligence and Photography Lab in the Department of Computer Science at Yonsei University. In particular, they forged and replaced persons’ facial marks such as eyes and mouth into other different person faces. Thus, such images are manipulated and considered as fake instances. The utilized dataset includes images, which vary from easy, mid, and hard recognition difficulty. The training dataset constitutes 924 and 973, while the testing dataset constitutes 121 and 108 “*Fake*” and “*Real*” labeled face images, respectively. All images had initial resolution of 600×600. This dataset is available at: https://www.kaggle.com/ciplab/real-and-fake-face-detection (accessed on 14 November 2021).

## 4. Experimental Results

In this section, we validate the efficiency and robustness of the proposed topology by performing comprehensive experimental simulations utilizing various state of the art CNN topologies. The measurement of quality is based on the well-known widely used evaluation metrics: Accuracy (Acc), Geometric Mean (GM), and the Area Under the Curve (AUC) [41]. Notice that the performance metrics GM and AUC present the information provided by a confusion matrix in compact form [42,43]; hence, these two metrics constitute the proper ones to evaluate if a prediction model has not overfitted the training data. The best performance for each state-of-the-art model and performance metric is highlighted in bold. The implementation code was written in Python 3.7 utilizing FastAI library [44], while the hyper-parameters were defined under exhaustive experimentation.

In our implementation, the cost function (1) was minimized utilizing Adam optimization algorithm [29], and the value of the learning rate was set to $10^{-3}$. Additionally, in order to avoid overfitting and maximize the efficiency of the proposed CAE, 10% of training data was used for validation and early stopping technique based on “validation loss’’ was used.

The evaluated approaches are the “Traditional”, “Means Denoising (MD)”, and the proposed one, “CAE”. In the Traditional approach, the images are directly fitted to a CNN classification model, while in the MD approach, a non-local MD algorithm [45] was applied on every input image, as a first preprocessing step, before feeding the images into the CNN classification models. The motivation behind this approach was to provide a more comprehensive experimental comparison by evaluating the proposed methodology against a classical approach which uses an image processing denoising technique. Finally, in our proposed CAE approach, the images are initially compressed and transformed via the CAE model and then fitted to a CNN classification model.

Table 2, Table 3 and Table 4 summarize the performance of the evaluated approaches regarding plant disease, skin cancer, and DeepFake experts, respectively. Notice that each approach has been evaluated utilizing VGG, ResNet, DenseNet, and MobileNet as a pre-trained CNN classification model. Notice that all pre-trained models were trained on ImageNet dataset. The interpretation of Table 2, Table 3 and Table 4 reveals that the incorporation of CAE managed to significantly increase the performance of all CNN models in all case study scenarios, especially for the VGG model in which is observed a considerable improvement. In addition, it managed to outperform the MD image pre-processing technique for every utilized pre-trained CNN. More specifically, the proposed CAE approach managed to significantly increase the performance of the ResNet, DenseNet, and MobileNet CNN model producing the best results for all datasets. Moreover, it managed to considerably increase the performance of the VGG model for all utilized datasets. Furthermore, the DenseNet CNN model managed to deliver the best results overall for all utilized approaches (Traditional, MD, CAE) and all utilized datasets, while the VGG model reported the worst results. However, utilizing the proposed CAE approach, the VGG model managed to achieve a decent and great performance, similar to the other CNN models.

Finally, it is worth mentioning that in our preliminary experiments, we fitted the CNN models with the original images, and we utilized the reconstructed images on the testing phase. However, the performance of all models was similar or slightly degraded.

Another significant finding is that the incorporation of CAE considerably improved the performance of the CNN models in the DeepFake Experts dataset. This is probably due to the fact that this dataset is considered as a very noisy dataset with considerable amount of redundant information in every image. This means that the CAE managed to capture the relevant information (fake signs on every face for this case study scenario), filtering out the noise. Therefore, the CNN classification models had to focus only on the most relevant information, thus leading to this great performance improvement.

Clearly, the purpose of this study was not to propose a deep learning classifier but to demonstrate a complete topology for addressing hard image classification problems in which the training image data has a high-dimensional size and contains noise. As a result, it obviously leads to some computational cost increase for training the CAE, but on the other hand, reducing the noise leads to a considerable reduction in computational cost for training the DL model (smaller figures).

To summarize, our results demonstrated that the incorporation of convolutional autoencoders as an image preprocessing technique could improve the performance of CNN models leading to robust and accurate results. Therefore, it can be considered as a promising tool on high-dimensional and noisy dataset applications.

Next, we attempt to provide statistical evidences about the efficiency of our proposed approach. More analytically, we investigate if the hypothesis $H_{0}$ that all evaluated approaches, i.e., “Traditional”, “Means Denoising (MD)”, and the proposed one, “CAE”, performed equally well for a given level. For this purpose, we used the non-parametric Friedman Aligned Ranking (FAR) [46] test. Furthermore, for examining if the differences in the performance of the utilized pre-trained CNN models are statistically significant, we applied the post hoc Finner test [47] with significance level α=5%.

Table 5, Table 6 and Table 7 report the statistical analysis, performed by nonparametric multiple comparison, relative to Accuracy, GM, and AUC performance metrics, respectively. Clearly, the results presented in Table 5, Table 6 and Table 7 provide statistical evidence that the proposed approach reported the highest probability-based ranking, outperforming the other approaches.

## 5. Conclusions

In this work, we proposed and suggested the incorporation of convolutional autoencoders as a general unsupervised learning data preprocessing method for creating robust and compressed feature representations in order to improve CNN performance on image classification tasks.

The utilized CNN models (ResNet, DenseNet, MobileNet, and VGG) are generally considered as widely used state-of-the-art (SoA) image classification models. Furthermore, our scope in this research work is not to prove directly that our method leads to the highest performance results comparing with other SoA image classification models. In contrast, our aim is to prove that the proposed method is able to improve any SoA CNN model via the combination and incorporation of the CAE component. Therefore, we consider that the utilization of four SoA image classification models is a sufficient number in model selection in order to lead to reliable and robust experimental results.

In order to validate the efficiency of the proposed methodology, we utilized datasets from three very popular and totally different application domains, the plant disease, skin cancer, and DeepFake detection problems, applying state-of-the-art CNN model architectures such as ResNet and DenseNet. Based on our experimental results, the proposed methodology was significantly superior compared to every other utilized approach. Note that it is possible that the prediction ability of the proposed approach could be further improved by including more sophisticated DL tools, loss and activation functions, class weighted approaches, etc. (see [48] and the references therein). This is to be included and fully investigated in our future research.

Nevertheless, the limitation of the proposed framework lies in the fact that there is no mathematical proof that it outperforms traditional methodologies nor that the conditions of the Nyquist theorem are satisfied. The conclusions and findings of this work can be demonstrated only from experimental and qualitative reasoning. Furthermore, the utilized real-world datasets did not provide us with any information about the distribution and the kind of noise. To this end, we were not able to estimate the expense of error or the image quality improvement. These also constitute general limitations to all prior works [8,15,23,24], which proposed denoising schemes based on the use of CAEs.

Additionally, CAEs are usually prone to overfitting which implies that in such cases, they may not create high-quality data for fitting a Deep Learning model. From our numerical experiments, we have not noticed such cases; however, more experiments are needed. This is probably dependent on the problem at hand and the quality of the original images.

Another limitation of the proposed work is that the level of compression is dependent on the CAE’s architecture and more specifically on the output of the encoder. Note that the smaller the output of the encoder, the higher the level compression. However, for identifying the optimal level of compression, we believe that more experimentation is needed in order to examine and evaluate the performance of the proposed approach using different architectures and eventually different levels of compression.

In future work, we aim to investigate ensemble learning methods [49,50,51,52] such as Bagging and Stacking and combine them with our proposed topology, aiming to create more robust and accurate composite classification models. In addition, we intend to explore further the efficacy and efficiency of the proposed network in a variety of datasets of various sizes and complexity from real-world application domains. The vigorous development of the Internet and the widespread adoption of electronic medical records have led to the development of large repositories of labeled and mostly of unlabeled biomedical images. Nevertheless, the process of correctly labeling new, unlabeled instances frequently requires the efforts of specialized personnel, which incur a lot of time and high monetary costs. To deal with this problem, Semi-Supervised Learning algorithms constitute the appropriate machine learning methodology for extracting useful knowledge from both labeled and unlabeled data in order to build efficient prediction models. To this end, another interesting idea could be the adoption of the proposed autoencoder methodology to Semi-Supervised Learning (SSL) techniques [53] for addressing challenging biomedical classification tasks. SSL is a new state-of-the-art data mining area, which focuses on applications where the labeled data are limited and require much effort and cost to obtain. Since the autoencoders do not require by default labeled data for their training procedure, the incorporation of the proposed framework in the SSL area could provide us with promising models. Finally, we also aim to incorporate intrinsic interpretability using explainable features [54,55,56] in order to provide some degree of explainability.

## Figures and Tables

**Table 1:** Parameter settings of the utilized CAE topology.

	Layers	Input Size	Kernel Size	Stride	Output Size
Encoder	2D Conv (E. Input)	H×W×3	4×4×32	2×2×1	H/2×W/2×32
	ReLU	H/2×W/2×32	−	−	H/2×W/2×32
	2D Conv	H/2×W/2×32	2×2×3	2×2×1	H/4×W/4×3
	Sigmoid (E. Output )	H/4×W/4×3	−	−	H/4×W/4×3
Decoder	2D Deconv3 (D. Input)	H/4×W/4×3	2×2×32	2×2×1	H/2×W/2×32
	ReLU	H/2×W/2×32	−	−	H/2×W/2×32
	2D Deconv4	H/2×W/2×32	4×4×3	2×2×1	H×W×3
	Sigmoid (D. Output)	H×W×3	−	−	H×W×3

**Table 2:** Performance results for plant disease benchmark. The best results are displayed in bold.

Approaches	CNN Model	Acc	GM	AUC
Traditional		80.0%	852	0.866
MD	VGG	83.2%	906	0.896
CAE		**87.6%**	**913**	**0.917**
Traditional		90.3%	1027	0.935
MD	ResNet	91.4%	1044	0.946
CAE		**92.4%**	**1066**	**0.949**
Traditional		93.0%	1187	0.953
MD	DenseNet	92.9%	**1188**	0.954
CAE		**93.5%**	1140	**0.956**
Traditional		**94.6%**	1031	**0.963**
MD	MobileNet	92.9%	1075	0.957
CAE		93.5%	**1087**	0.956

**Table 3:** Performance results for skin cancer benchmark. The best results are displayed in bold.

Approaches	CNN Model	Acc	GM	AUC
Traditional		66.3%	38	0.631
MD	VGG	67.4%	39	0.637
CAE		**70.0%**	**40**	**0.750**
Traditional		73.7%	37	0.633
MD	ResNet	74.7%	39	0.642
CAE		**77.9%**	**42**	**0.697**
Traditional		75.8%	**42**	0.685
MD	DenseNet	75.3%	41	0.681
CAE		**80.0%**	41	**0.689**
Traditional		**75.2%**	35	0.603
MD	MobileNet	74.2%	36	0.612
CAE		72.0%	**39**	**0.638**

**Table 4:** Performance results for DeepFake experts benchmark. The best results are displayed in bold.

Approaches	CNN Model	Acc	GM	AUC
Traditional		73.3%	83	0.732
MD	VGG	76.4%	87	0.774
CAE		**82.0%**	**93**	**0.869**
Traditional		80.8%	92	0.881
MD	ResNet	81.2%	93	0.892
CAE		**82.1%**	**94**	**0.897**
Traditional		84.3%	96	0.905
MD	DenseNet	83.4%	95	0.903
CAE		**86.1%**	**98**	**0.926**
Traditional		79.0%	90	0.853
MD	MobileNet	79.5%	91	0.857
CAE		**81.0%**	**92**	**0.889**

**Table 5:** FAR test and Finner post hoc test based on Accuracy metric.

Series	Friedman	Finner Post Hoc Test	
	Ranking	*p*-Value	$H_{0}$
CAE	9.4167	−	−
MD	21.6667	0.004399	Rejected
Traditional	24.4167	0.000975	Rejected

**Table 6:** FAR test and Finner post hoc test based on GM metric.

Series	Friedman	Finner Post Hoc Test	
	Ranking	*p*-Value	$H_{0}$
CAE	11.5000	−	−
MD	17.8333	0.140894	Not rejected
Traditional	26.1667	0.00065	Rejected

**Table 7:** FAR test and Finner post hoc test based on AUC metric.

Series	Friedman	Finner Post Hoc Test	
	Ranking	*p*-Value	$H_{0}$
CAE	7.58330	−	−
MD	22.0417	0.000775	Rejected
Traditional	25.8750	0.000021	Rejected

## Data Availability

Not applicable.

## List of Acronyms and Abbreviations

Acc	Accuracy
AUC	Area under the curve
CAE	Convolutional autoencoders
CNN	Convolutional neural networks
CAE-CNN	Convolutional autoencoder–convolutional neural network
DL	Deep learning
GCRF	Gaussian conditional random field
GM	Geometric mean
GM-MRF	Gaussian mixture Markov random field model
GSM	Gaussian scale mixture
ISIC	International skin imaging collaboration
LSS	Least squares solution
MD	Means denoising
ML	Machine learning
MRF	Markov random fields
